# Impacts on Global Health from Nursing Research

**DOI:** 10.4269/ajtmh.16-0918

**Published:** 2017-04-05

**Authors:** Kimberly Baltzell, Monica McLemore, Mona Shattell, Sally Rankin

**Affiliations:** 1 Center for Global Health, University of California San Francisco, San Francisco, California; 2 Department of Family Health Care Nursing, University of California San Francisco, San Francisco, California; 3 Department of Community, Systems, and Mental Health Nursing, Rush University, Chicago, Illinois

## Abstract

Infectious disease continues to adversely affect populations in low- and middle-income countries. Investments in solutions often focus on technology, yet health-care workers remain in short supply. Nurses are the largest cadre of health-care workers and are largely responsible for patient care around the world. In fact, it is estimated that nurses care for nine out of every 10 patients seen. Importantly, sound nursing science contributes to solutions that directly impact patient care, especially those that pertain to infectious disease. Here we share several examples of nursing science that are improving care delivery in three global health areas: human immunodeficiency virus testing and prevention strategies in Malawi, family planning in Kenya, and response to Ebola virus disease.

Often referred to as the “backbone of health-care systems,” nurses are key for the detection, treatment, and prevention of infectious disease in many settings. In fact, nurses are responsible for the care of nine of 10 patients worldwide. Over a century ago, Florence Nightingale recognized both the need for formal training for nurses as well as the power of the nurse to improve patient outcomes. She was considered an early and brilliant pioneer in public health, epidemiology, and infectious disease research when she observed that British soldiers in the Crimean War were more likely to die of infection than battle injuries.^1^

In the 21st century, nurses are key in the prevention of infection. For example, nurses deliver 80% of all babies worldwide and their meticulous attention to infection control during delivery prevents countless neonatal infections. Likewise, nurses in lower and middle income countries, as well as more affluent settings, prevent surgical infections and iatrogenic infections in wards, operating rooms, and emergency settings. In addition, nurses are frontline health-care providers in community settings, and their attention to problems of infection control in under-resourced community settings cements their importance in the workforce.

While many agree that well-trained nurses are critical for the actual delivery of health care, what is less well known are the unique contributions of nurses in creating solutions in health-care delivery. A recent blog post in *Scientific American* highlights important research by nurses, from exploring links to Alzheimer's to human papilloma virus and cancer.^2^ Moreover, the National Institute of Nursing Research emphasizes the importance of a global focus in achieving its mission of “advancing nursing science to improve the health and well-being of all the world's citizens.”^3^ Importantly, nurse scientists are trained to evaluate social, economic, and psychological, as well as biological, aspects to causes of disease, disease management, and prevention, making them uniquely qualified to develop global health solutions with staying power.

The purpose of this perspective is to show how nursing research and nursing practice provide global health solutions. We will describe work in three global health areas where ideas were originated and sustained by nurse researchers and nurse-led health-care teams—human immunodeficiency virus (HIV) testing and prevention strategies in Malawi, family planning in Kenya, and response to the recent Ebola outbreak.

## HIV Testing and Prevention

In Malawi, nursing research created unconventional solutions for engaging patients in HIV testing. Although some of these solutions were technical in nature, others addressed social determinants of health, such as understanding the influence of religious leaders on promotion of HIV testing and prevention behaviors.^4^ This type of understanding is necessary to lower transmission rates, prevent spread within families, and reduce the number of orphans needing care. For example, nursing research led to the successful implementation of peer group leaders in high-risk communities. These group leaders were responsible for educating populations on HIV prevention. The study found peer group leaders remained in their roles long after the study period, giving communities a strategy for HIV prevention in the absence of a robust health-care structure.^5^ Nursing research uncovered successful strategies for nurse workforce retention rates in Malawi^6^ and evaluated successful strategies for HIV prevention among health-care workers, both demonstrating the importance of nursing research for improving population health and workforce capacity resulting in access to quality care.

## Family Planning

Although family planning is important to women's health for many reasons, its role in the control and mitigation of the impact of infectious disease cannot be ignored. For many women in Kenya and other sub-Saharan countries, coinfection of HIV and malaria contributes to a variety of poor reproductive outcomes including preterm birth, hemorrhage, and maternal mortality. The need for family planning is immense in Kenya given the rate of contraception use in reproductive-aged individuals ranges from 28% to 34%.^7^,^8^ Family planning services also provide childbearing families the opportunity to space their pregnancy to support optimal health outcomes for pregnant women. Innovative nurse-led interventions are necessary, as they are shown to improve reproductive health outcomes. For example, in Kenya, nurses are currently leading novel work to integrate family planning across the antenatal, perinatal, and postnatal continuum for childbearing families.^9^ In this project, nurses serve as champions to support and partner with community health workers to provide care to newly postpartum individuals. Satisfaction with this program is high, and family planning service utilization is more consistent in participants in this program than in the broader population of Kenyan women.

## Ebola

In the recent Ebola outbreak in west Africa, survival depended on two key elements—early detection and nursing care once diagnosed. Nurses were key for preventing additional infections, saving those who were infected, and comforting families of those who did not survive, all while working in understaffed facilities, an unfortunate but ordinary circumstance in low- and middle-income countries.^10^ In fact, nurses outnumbered all other groups of health-care workers delivering care during the outbreak in West Africa. As numerous health-care workers were infected during the outbreak, it was nursing that challenged the personal protective equipment protocol recommended by the Centers for Disease Control and Prevention. Based on nursing input, protocols and levels of protection were improved to protect health workers.^11^ It is impossible to calculate the number of health worker lives that were saved as a result of this nursing science.

These are just a few examples of solution-driven work that is grounded in rigorous evidence developed for and by nurses. While infectious disease remains a major driver of poor health around the world, nurse scientists will continue adding to the body of knowledge on how best to prevent infection and improve patient outcomes.
